# Screening of key genes responsible for *Pennisetum setaceum* ‘Rubrum’ leaf color using transcriptome sequencing

**DOI:** 10.1371/journal.pone.0242618

**Published:** 2020-11-23

**Authors:** Ting Zhu, Xia Wang, Zhimin Xu, Jie Xu, Rui Li, Ning Liu, Guochang Ding, Shunzhao Sui

**Affiliations:** 1 College of Arts College of Landscape Architecture, Fujian Agriculture and Forestry University, Fuzhou, China; 2 College of Horticulture and Landscape Architecture, Southwest University, Chongqing, China; Hainan University, CHINA

## Abstract

*Pennisetum setaceum* ‘Rubrum’ is an ornamental grass plant that produces purple leaves in high-light environments and light purple or green leaves in low-light environments, the latter of which greatly reduces its aesthetic appeal. Therefore, we aimed to identify the key genes associated with leaf coloration and elucidate the molecular mechanisms involved in the color changes in *P*. *setaceum* ‘Rubrum’ leaves. We performed transcriptome sequencing of *P*. *setaceum* ‘Rubrum’ leaves before and after shading. A total of 19,043 differentially expressed genes were identified, and the numbers of upregulated and downregulated genes at T1 stage, when compared with their expression at the T0 stage, were 10,761 and 8,642, respectively. The possible pathways that determine *P*. *setaceum* ‘Rubrum’ leaf color included flavonoid biosynthesis, flavone and flavonol biosynthesis, and carotenoid biosynthesis. There were 31 differentially expressed genes related to chlorophyll metabolism, of which 21 were related to chlorophyll biosynthesis and 10 to chlorophyll degradation, as well as three transcription factors that may be involved in the regulation of chlorophyll degradation. There were 31 key enzyme genes involved in anthocyanin synthesis and accumulation in *P*. *setaceum* ‘Rubrum’ leaves, with four transcription factors that may be involved in the regulation of anthocyanin metabolism. The transcriptome data were verified and confirmed reliable by real-time fluorescence quantitative PCR analysis. These findings provide a genetic basis for improving leaf color in *P*. *setaceum* ‘Rubrum.’

## Introduction

In recent years, ornamental grass with colorful leaves and novel morphologies has become popular for landscaping and gardening. Owing to its strong ecological adaptability and unique artistic use, ornamental grass adds freshness to existing urban plant design. Ornamental grass with colored leaves, especially deep reds, purples, and other warm tones can create a mysterious or cozy atmosphere [1]. *P*. *setaceum* ‘Rubrum’ is a cultivated grass with an elegant shape and light and delicate panicles. Its natural and simple appearance brings unique beauty and draws attention to the landscaping design. Plant color, as an essential factor of sensory quality, has a noticeable effect on ornamental value and commercial value [2]. Thus, the purplish-red leaves of *P*. *setaceum* ‘Rubrum’ contribute to its value; however, in our previous study, we show that when *P*. *setaceum* ‘Rubrum’ is grown in the shade, its leaf color turns green and the pigment content changes [3].

Chlorophyll and anthocyanins are the primary pigments that determine plant leaf color. Chlorophyll gives leaves their green color, while anthocyanin is the primary pigment responsible for red, purple, and blue colors [4]. Chloroplasts produce various pigments, including carotenoids and chlorophyll, which are the main pigment components in regular green leaves [4]. At present, most research has been mainly focused on the biosynthesis and degradation of chlorophyll. In the model plant *Arabidopsis thaliana*, 27 genes encode the 15 enzymes involved in chlorophyll biosynthesis from glutamyl-tRNA(Glu) to chlorophyll *b* [5]. The chlorophyll biosynthesis pathway starts with glutamyl-tRNA, progresses through a series of reactions catalyzed by glutamyl-tRNA reductase (HemA), Mg-chelatase, light-dependent NADPH: rotochlorophyllide oxidoreductase (POR), chlorophyllide *a* oxygenase (CAO), and other enzymes before it ends with chlorophyll *a* and chlorophyll *b* as products [5, 6]. The anthocyanin biosynthesis pathway is a branch of the flavonoid biosynthesis pathway in many plants [7], and most of the genes in this pathway have been identified and cloned [8]. The main enzymes related to anthocyanin biosynthesis are phenylalanine ammonia-lyase (PAL), chalcone isomerase (CHI), dihydroflavonol-4-reductase (DFR), and UDPG-flavonoid 3-O-glucosyltransferase (UFGT). Among these, PAL is a key enzyme in the metabolic pathway of phenylpropionic acid in plants and plays a significant role in the biosynthesis of lignin and flavonoids [9]. CHI catalyzes the stereospecific and intramolecular isomerization of naringenin chalcone into corresponding (2S)-flavanones, and improves the conversion rate of the isomerization reaction [10]. DFR can reduce dihydrokaempferol to produce leucopelargonidin [11, 12], and UFGT, which is the last enzyme in anthocyanin biosynthesis, converts unstable anthocyanins into stable anthocyanins [13]. Transcription factors (TFs) involved in the anthocyanin biosynthetic pathway mainly belong to the three protein families, R2R3-MYB, basic helix loop helix (bHLH), and WD40 repeat (WDR) [14, 15]. Anthocyanin biosynthesis is mainly regulated by the interaction between two or three of these proteins [14–16].

Among the external environmental factors, light intensity is the most crucial factor affecting leaf color in plants [17]. Under different light intensities and light durations, the contents of photosynthetic pigments and anthocyanins in plant leaves change, thus affecting the color of leaves. There have been reports on the effects of light on the color of a model plant, fruits, and several woody plants. For example, in the model plant *Petunia*, anthocyanin synthesis was repressed under shade conditions [18]. In fruit studies, light significantly increased the accumulation of flavonoids and the expression of biosynthetic genes in grape skin, while dark treatment decreased the accumulation of flavonoids in grape skin [19, 20]. Studies on woody plants of crabapple showed that prolonged sunlight exposure was beneficial to the accumulation of anthocyanins in the leaves of crabapple trees, resulting in red coloration [21]. The effect of light on anthocyanin accumulation at the molecular level is concentrated in model plants, while the mechanism of anthocyanin accumulation and red leaf coloration in most woody and herbaceous plants remains elusive [21]. Beckwith et al. used high-performance liquid chromatography and nuclear magnetic resonance to study the effects of different light intensities and various light sources on anthocyanin accumulation in the leaves of *P*. *setaceum* ‘Rubrum’ [2]; nonetheless, the molecular mechanisms of these correlations are rarely explored.

In our previous studies, we found that shading followed by light treatment significantly increased the values of the leaf color parameters L* (lightness) and b* (red-greenness), while a* values (blue-yellowness) decreased [3]. The physiological indexes chlorophyll *a* and chlorophyll *b* content significantly increased, whereas anthocyanin, flavonoid, soluble sugar content, and PAL and CHI activities significantly decreased [3]. We also found that the activities of PPO and POD did not change significantly [3]. This work indicated that light conditions could alter both chlorophyll and anthocyanin biosynthesis, although the molecular mechanism of leaf color changes still remains unclear. Therefore, the aims of the present study were to elucidate the molecular mechanisms that regulate leaf color parameters and physiological indicators. We performed transcriptome sequencing of leaves before and after shading and screened for key genes that control leaf coloration. This study will facilitate the horticulture and ornamental quality of *P*. *setaceum* ‘Rubrum’ and may also provide valuable information for further analysis of the molecular mechanisms involved in leaf coloration in herbaceous plant species.

## Materials and methods

### Plant materials and treatments

**Fig 1:**
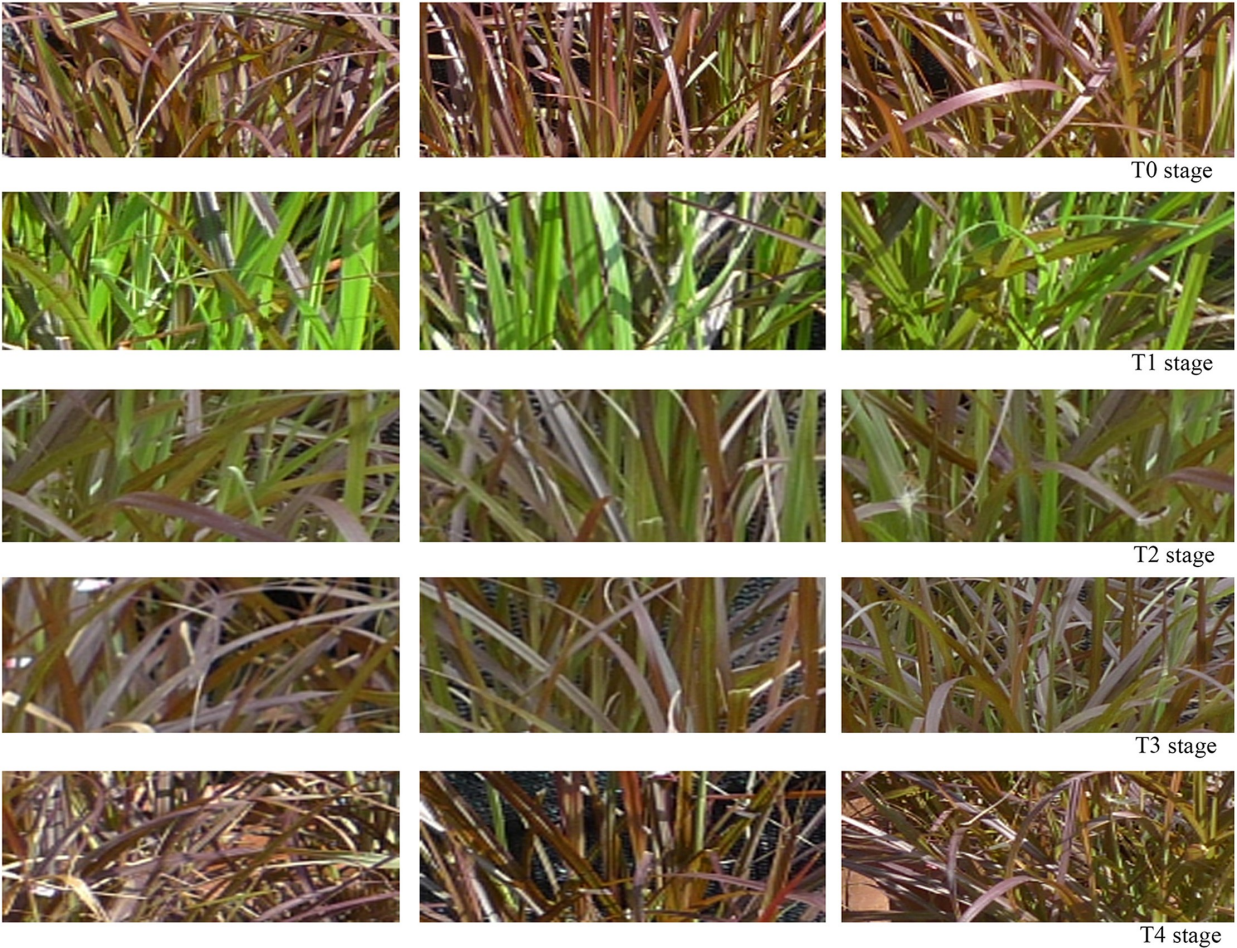
Dynamic changes of leaf color of *Pennisetum setaceum* ‘Rubrum’ in different stages. The three pictures in each row are three different plants randomly selected in each stage.

Seedlings of *P*. *setaceum* ‘Rubrum’ were transplanted from the ornamental grass base in Minqing County (Fuzhou City, Fujian Province) to the experimental base of Fujian Agriculture and Forestry University (Fujian Province, China) in mid-March 2018 and cultivated in open flower pots. The experiment was conducted in late June which is in the summer season for the region. The plants were shaded with a sunshade net that had a 10% transmittance, which was measured with an illuminance meter (transmittance = indoor illuminance/open—air illuminance). After the new leaves turned completely green, the sunshade net was removed and the plants were exposed to light until the leaf color returned to a purple color. On the basis of the leaf color change in the pre-experiment (Fig 1) [3], we selected T0, T1, T2, T3, and T4 time points to determine the leaf color parameters and physiological indexes: T0, untreated state; T1, stage when new leaves became completely green after 12 days of shading; T2, one day after the sunshade nets were removed; T3, three days after the sunshade nets were removed; and T4, stage at which the leaf color completely reverted to purple after 6 days in full light. The samples collected at stages T0 and T1 were used for transcriptome sequencing. To improve the reliability of the experimental results, the experiment was conducted in triplicate, with each experiment consisting of six pots. Leaf samples collected from the six pots were pooled together. The third or fourth leaf from the top of the plant was sampled at 9:00 every morning. The collected samples were immediately frozen in liquid nitrogen and stored at -80°C for further use.

### Experimental method

There were six mixed samples in the T0 and T1 groups. Total RNA was extracted using an RNAprep Pure Plant RNA Purification kit (Tiangen Biotech, Beijing, China) following the manufacturer’s instructions. The integrity of RNA was verified by RNase free agarose gel electrophoresis and the concentration was measured using an Agilent Biological Analyzer 2100 system (Agilent Technologies, Palo Alto, CA, USA). The Illumina Hi-Seq 4000 platform was used for sequencing, and the read length was 150 bp. Low-quality reads or reads containing adapter sequences were removed before bioinformatics analysis. The quality of transcriptome sequencing was confirmed by sequencing saturation and unigene coverage before conducting the next sequencing. Transcriptional group assembly was completed using Trinity software (http://trinityrnaseq.sf.net) [22]. First, the samples from the T0 and T1 stages were used to verify their reproducibility. A comprehensive analysis was performed using Principal Component Analysis and Pearson correlation coefficient (*r*^2^) as evaluation indices. Gene expression was calculated by reads per kilobase of transcript per million mapped reads (RPKM). Differentially expressed genes (DEGs) were screened according to the general filtering criteria of edgeR (|1og2Fold Change| > 1 and false discovery rate [FDR] < 0.05) [23]. The gene ontology analysis and Kyoto Encyclopedia of Genes and Genomes (KEGG) pathway enrichment analysis of DEGs in the T0 and T1 stages were based on the methods described by Young et al. [24] and Wu et al. [25], respectively, with a q ≤ 0.05 threshold value. Transcription factors were predicted based on PlantTFDB (http://planttfdb.cbi.pku.edu.cn) [26]. Ten key DEGs related to leaf color and two internal reference genes were selected for further analysis, and the reliability of the transcriptome results was verified by real-time quantitative PCR (qPCR) analysis [27]. Sequencing data are uploaded to the NCBI repository under BioProject PRJNA669318 (http://www.ncbi.nlm.nih.gov/bioproject/669318).

## Results

### Transcriptomic analysis of purple and green *P*. *setaceum* ‘Rubrum’ leaves

**Fig 2:**
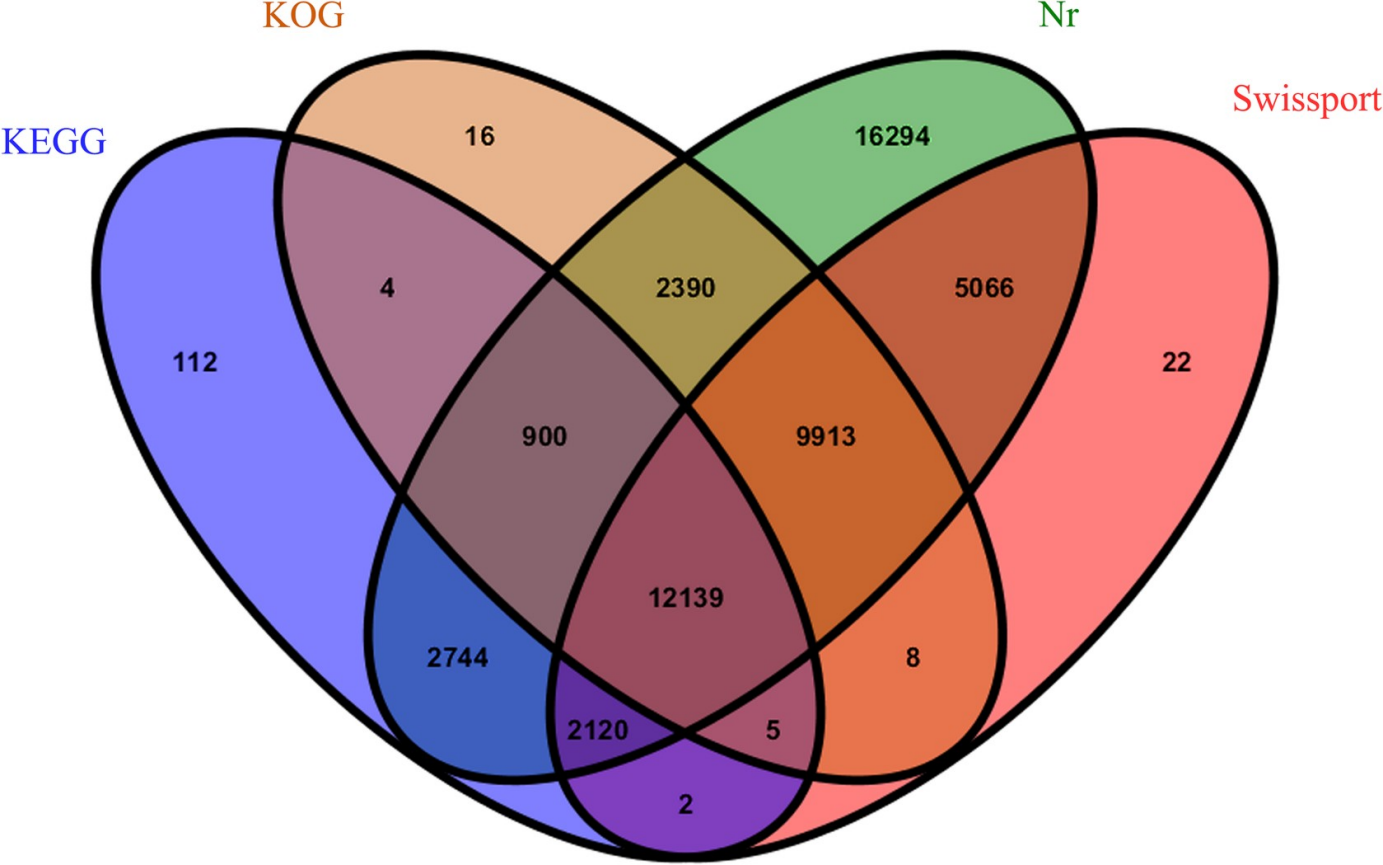
Venn diagram showing the number of genes annotated by four databases. Numbers indicate annotated unigenes.

The average Q20 and Q30 scores of RNA from samples collected in the T0 and T1 stages and sequenced on the Illumina Hi-Seq platform after filtration were greater than 98.35% and 94.82%, respectively, and had a GC content of approximately 56.20% (Table 1). The sequencing saturation (S1 Fig) and gene coverage (S2 Fig) results of each sample confirmed the good quality of this transcriptome sequencing, and the next step could proceed. A total of 93,750 unigenes were obtained using Trinity software, with N50 of 1362 nt and the average length of 916 nt; the overall assembly effect was good (Table 2). A total of 51,735 genes were annotated using four databases (Fig 2), namely the NCBI nonredundant protein database (Nr), Clusters of EuKaryotic Orthologous Groups of proteins database (KOG), KEGG, and Swiss-Prot database. The annotation success rate was the highest in the Nr database (55.00%) and the lowest in the KEGG database (19.23%).

**Table 1:** Statistics of sequencing data quality from *Pennisetum setaceum* ‘Rubrum’ samples.

Sample	T0-1	T0-2	T0-3	T1-1	T1-2	T1-3
**Raw reads**	51,967,690	42,468,986	43,154,866	51,658,934	47,604,938	48,009,044
**Clean reads**	50,807,520	41,559,440	42,233,576	50,467,394	46,514,920	46,890,932
**Q20 (%)**	98.37%	98.42%	98.42%	98.35%	98.38%	98.37%
**Q30 (%)**	94.90%	95.01%	95.03%	94.82%	94.92%	94.87%
**GC content**	55.74%	55.86%	55.44%	56.67%	56.69%	56.80%

**Table 2:** Assembly result statistics of *Pennisetum setaceum* ‘Rubrum’.

Genes Num	GC percentage	N50 (nt)	Max length (nt)	Min length (nt)	Average length (nt)	Total assemble bases
93750	51.1266	1362	11925	201	916	85,958,553

### Identification of differentially expressed genes in purple and green *P*. *setaceum* ‘Rubrum’ leaves

**Fig 3:**
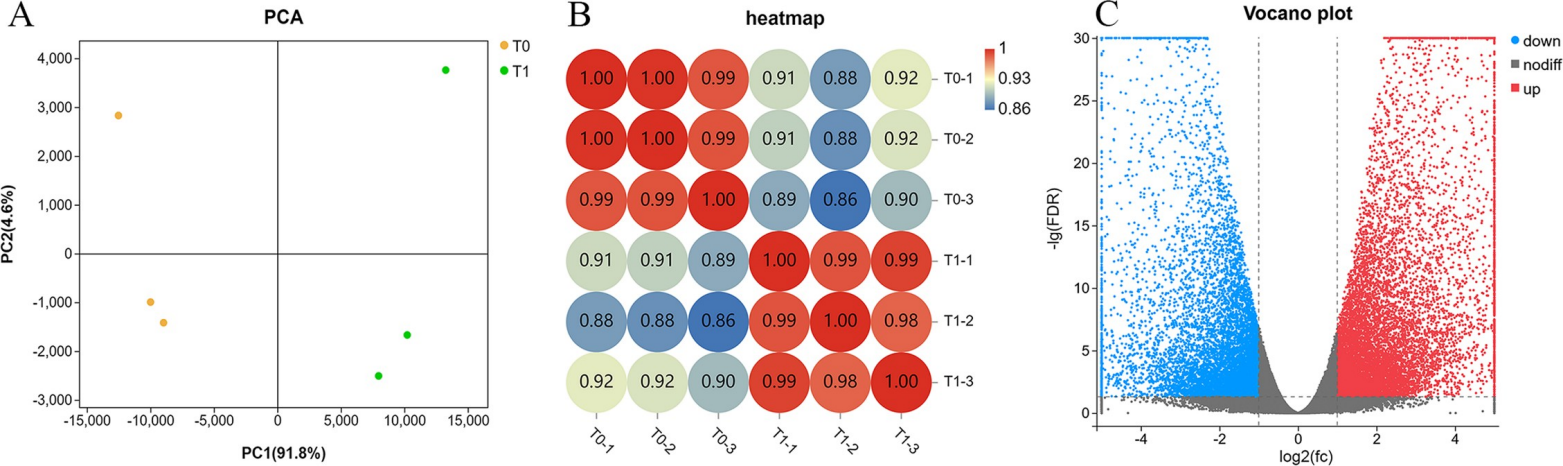
Sample relationship analysis and differentially expressed genes. (A) Principal components analysis. (B) *r*^*2*^ analysis to test repeatability. (C) Statistics of differentially expressed genes. Blue represents downregulated unigenes, and red represents upregulated unigenes.

Principal component and *r*^*2*^ analyses were used to evaluate sample repeatability and to test reliability. The first and second principal component explained 96.4% of the overall variance (Fig 3A), and the repeatability test showed a correlation greater than 0.98 between each sample (Fig 3B). The sample selection was reasonable, and the sample repeatability and test reliability were high. A volcano plot of 19,403 DEGs is presented in Fig 3C. There were 10,761 upregulated (red) and 8642 downregulated (blue) unigenes in T1 relative to their expression in T0.

### KEGG enrichment analysis identifies biosynthetic pathways responsible for leaf color

KEGG pathway analysis revealed that 9839 unigenes were associated with 131 pathways. A total of 386 unigenes were associated with metabolic pathways related to leaf color (Table 3). The main metabolic pathways related to leaf color were porphyrin and chlorophyll metabolism, photosynthesis, flavonoid biosynthesis, and carotenoid biosynthesis. The genes related to photosynthesis (27 genes) and anthocyanin (2 genes) were the least represented in this analysis.

**Table 3:** Leaf color-related metabolic pathways in *Pennisetum setaceum* ‘Rubrum’.

Number	Pathway	Gene count (9839)	Pathway ID
1	Porphyrin and chlorophyll metabolism	111 (0.62%)	ko00860
2	Photosynthesis	109 (0.60%)	ko00195
3	Flavonoid biosynthesis	71 (0.39%)	ko00941
4	Carotenoid biosynthesis	66 (0.37%)	ko00906
5	Photosynthesis-antenna proteins	27 (0.15%)	ko00196
6	Anthocyanin biosynthesis	2 (0.01%)	ko00942

**Fig 4:**
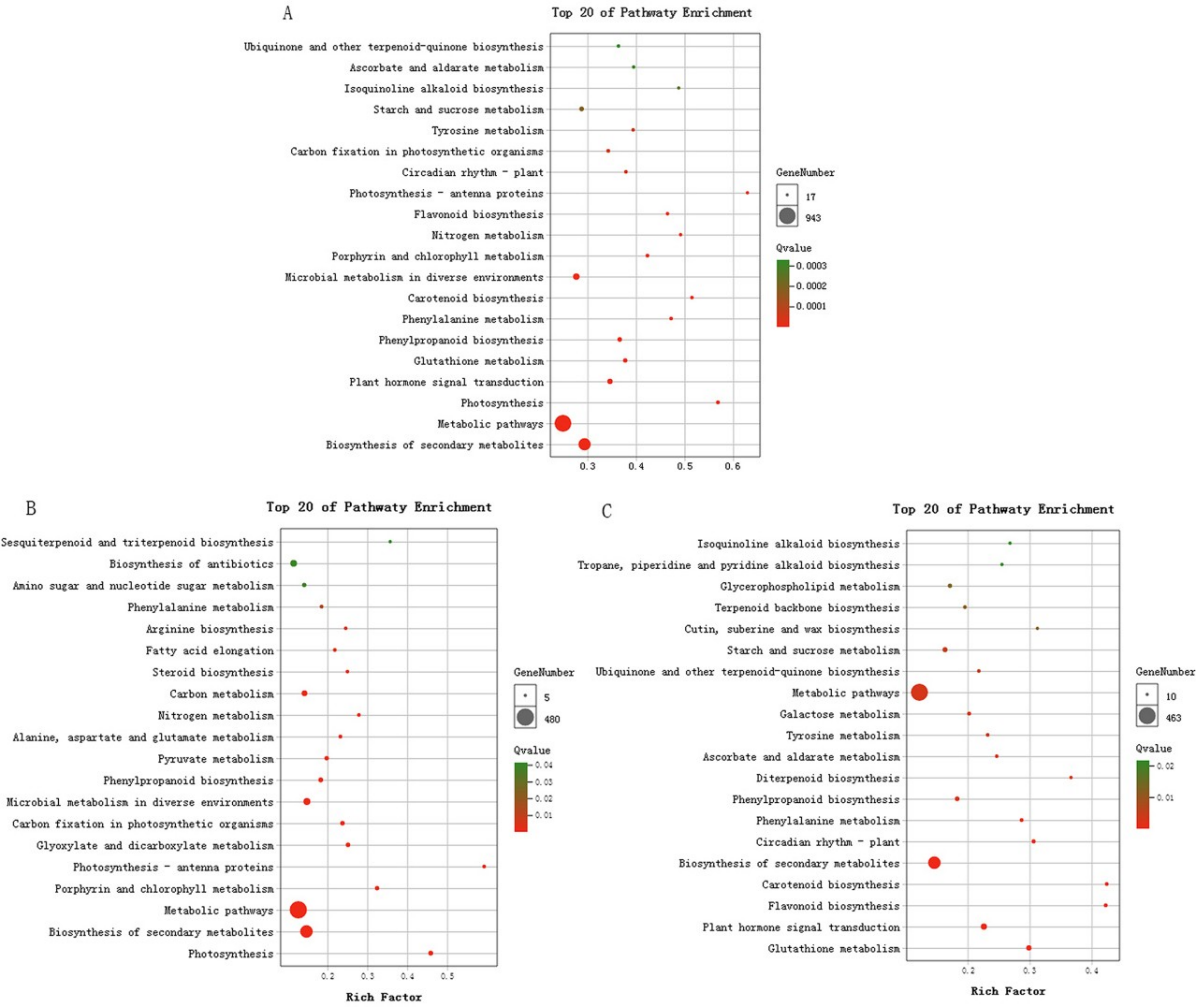
KEGG pathway enrichment of Differentially Expressed Genes (DEGs) in *Pennisetum setaceum* ‘Rubrum’. (A) All DEGs enriched in KEGG pathways; (B) Pathway enrichment of upregulated DEGs; (C) Pathway enrichment of downregulated DEGs.

A total of 1976 DEGs annotated in the KEGG database were significantly enriched in 34 pathways. Biosynthesis of secondary metabolites was the most significantly enriched pathway, followed by metabolic pathways and photosynthesis (Fig 4A). Among the pathways related to plant pigmentation, 33 DEGs were enriched in flavonoid biosynthesis pathway, four DEGs were in the flavone and flavonol biosynthesis pathway, 34 DEGs were in the carotenoid biosynthesis pathway, and two DEGs were in the anthocyanin biosynthesis pathway. These results suggested that leaf coloration in *P*. *setaceum* ‘Rubrum’ is associated with changes in these metabolic pathways.

Most of the DEGs that were upregulated in the T1 stage (Fig 4B) were enriched in the following pathways: photosynthesis, porphyrin and chlorophyll metabolism, photosynthesis-antenna proteins, and carbon fixation in photosynthetic organisms. Among the downregulated genes (Fig 4C), DEGs were significantly enriched in flavonoid biosynthesis, carotenoid biosynthesis, phenylalanine metabolism, and phenylpropanoid biosynthesis.

#### Genes related to chlorophyll metabolism

A previous study found that chlorophyll, anthocyanins, flavonoids, and PAL and CHI activities are closely related to leaf color [3]. Therefore, in the present study, we focused on KEGG database annotation and prediction of TFs to investigate the regulatory relationship of these structural genes and TFs on leaf color in *P*. *setaceum* ‘Rubrum.’

**Fig 5:**
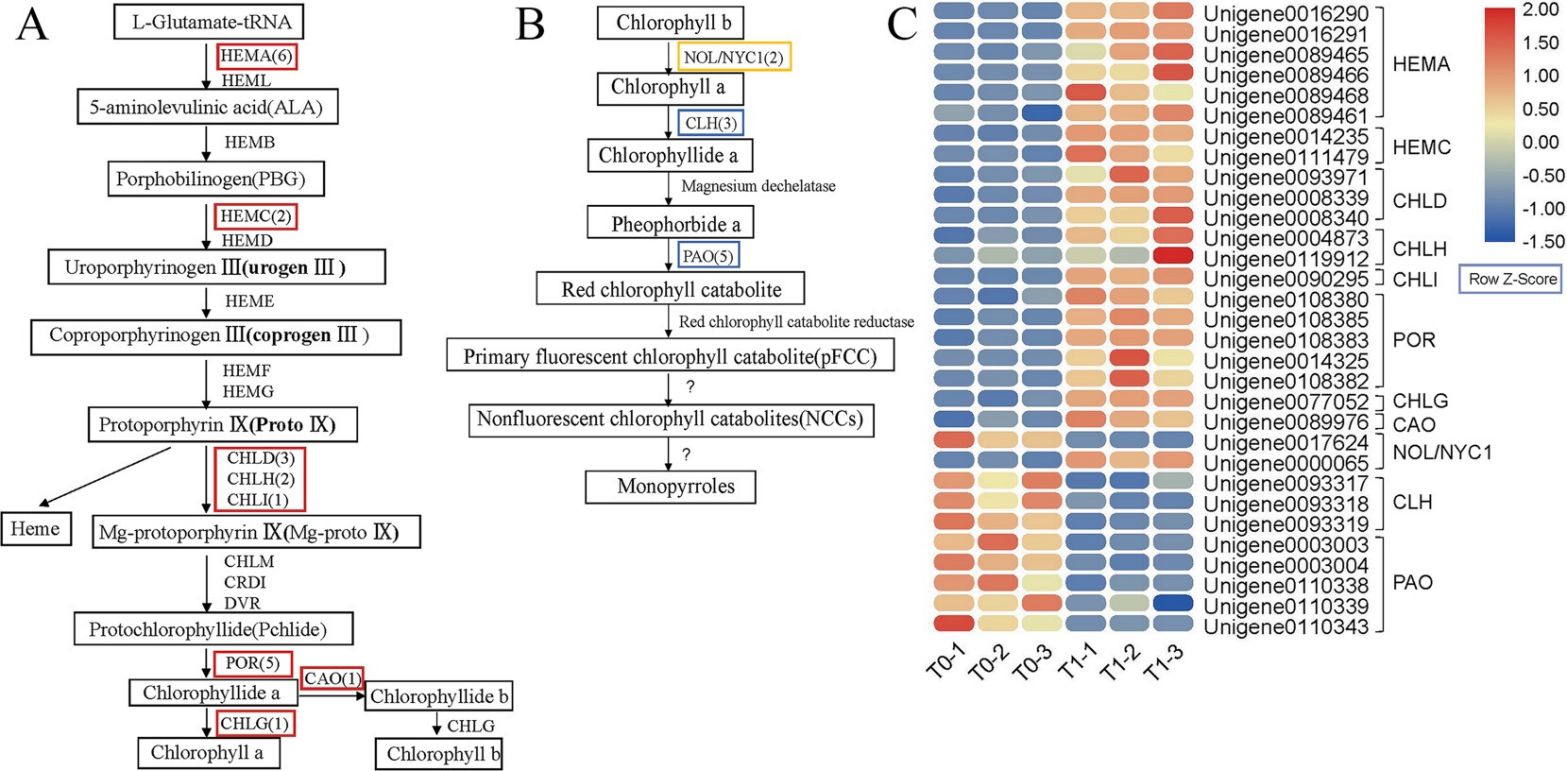
Chlorophyll metabolism pathway and heat map of *Pennisetum setaceum* ‘Rubrum’. (A) Chlorophyll biosynthesis pathway [5, 6], (B) chlorophyll degradation pathway [28–30], and (C) heat map of pathway-related differentially expressed genes. The red box indicates upregulated genes, the blue box indicates downregulated genes, and the orange box indicates genes that are both upregulated and downregulated in T1 stage.

Based on the pathway analysis of the DEGs, 31 DEGs encode enzymes that regulate chlorophyll metabolism (Fig 5A and 5B). In order to analyze the pathways modulated by chlorophyll-related DEGs, we analyzed chlorophyll metabolism pathway and heat map of *P*. *setaceum* ‘Rubrum’. Twenty-one unigenes were related to chlorophyll synthesis and 10 to chlorophyll degradation (Fig 5C). Among the DEGs involved in chlorophyll biosynthesis (Fig 5A), six genes were predicted to belong to the HemA enzyme family, which functions in the first stage of chlorophyll biosynthesis. In the second stage of chlorophyll biosynthesis, two porphobilinogen deaminase (HemC) genes were predicted to encode porphobilinogen (PBG). In the last stage of chlorophyll biosynthesis, three genes were annotated to the magnesium chelatase subunit ChlD, two genes to magnesium chelatase subunit ChlH, and one gene to magnesium-chelatase subunit ChlI of the Mg-chelatase enzyme. Five genes were annotated to *POR*, one gene was annotated to *chlorophyll synthase* (*ChlG*), and one gene was annotated to *CAO*. These DEGs are the key regulatory enzymes in chlorophyll biosynthesis and were upregulated at the T1 stage.

A gene annotated to *NON-YELLOWCOLORING1* (*NYC1*), which is related to chlorophyll degradation (Fig 5B), was downregulated, whereas a gene annotated to *NYC1-LIKE* (*NOL*) was upregulated in the T1 stage. There were three downregulated genes in the chlorophyllase (CLH) family. A total of five genes that were related to pheophorbide *a* oxygenase (PaO) were downregulated in the T1 stage.

Based on PlantTFDB [26], the annotation results for the TFs predicted five genes that regulate chlorophyll biosynthesis (Table 4). These five TFs were all annotated in the phytochrome interacting factors (PIFs) family. According to the gene annotation, as long as the genes coding helicase/helix were downregulated, the chlorophyll content in the T1 stage was higher than that in T0, indicating that the helicase encoded by these genes may lead to chlorophyll degradation.

**Table 4:** Differentially expressed transcription factors associated with chlorophyll biosynthesis in *Pennisetum setaceum* ‘Rubrum’.

Transcription factor family	Gene ID	log2 Ratio(T1/T0)	Symbol	Gene annotation
PIFs	Unigene0051109	-1.16	PIF1	PREDICTED: ATP-dependent DNA helicase PIF1-like [*Oryza sativa* Japonica group]
	Unigene0051114	-1.98	PIF1	PREDICTED: ATP-dependent DNA helicase PIF1-like [*Oryza sativa* Japonica group]
	Unigene0075348	-1.56	PIF1	helicase-like protein [*Oryza sativa* Japonica group]
	Unigene0031361	-2.10	PIF3	Helix-loop-helix DNA-binding domain containing protein [*Oryza sativa* Japonica group]
	Unigene0019008	-1.78	PIF6	ATP-dependent DNA helicase PIF6-like [*Aegilops tauschii* subsp. tauschii] [*Aegilops tauschii*]

#### Genes related to anthocyanin metabolism involved in *P*. *setaceum* ‘Rubrum’ leaf color

**Fig 6:**
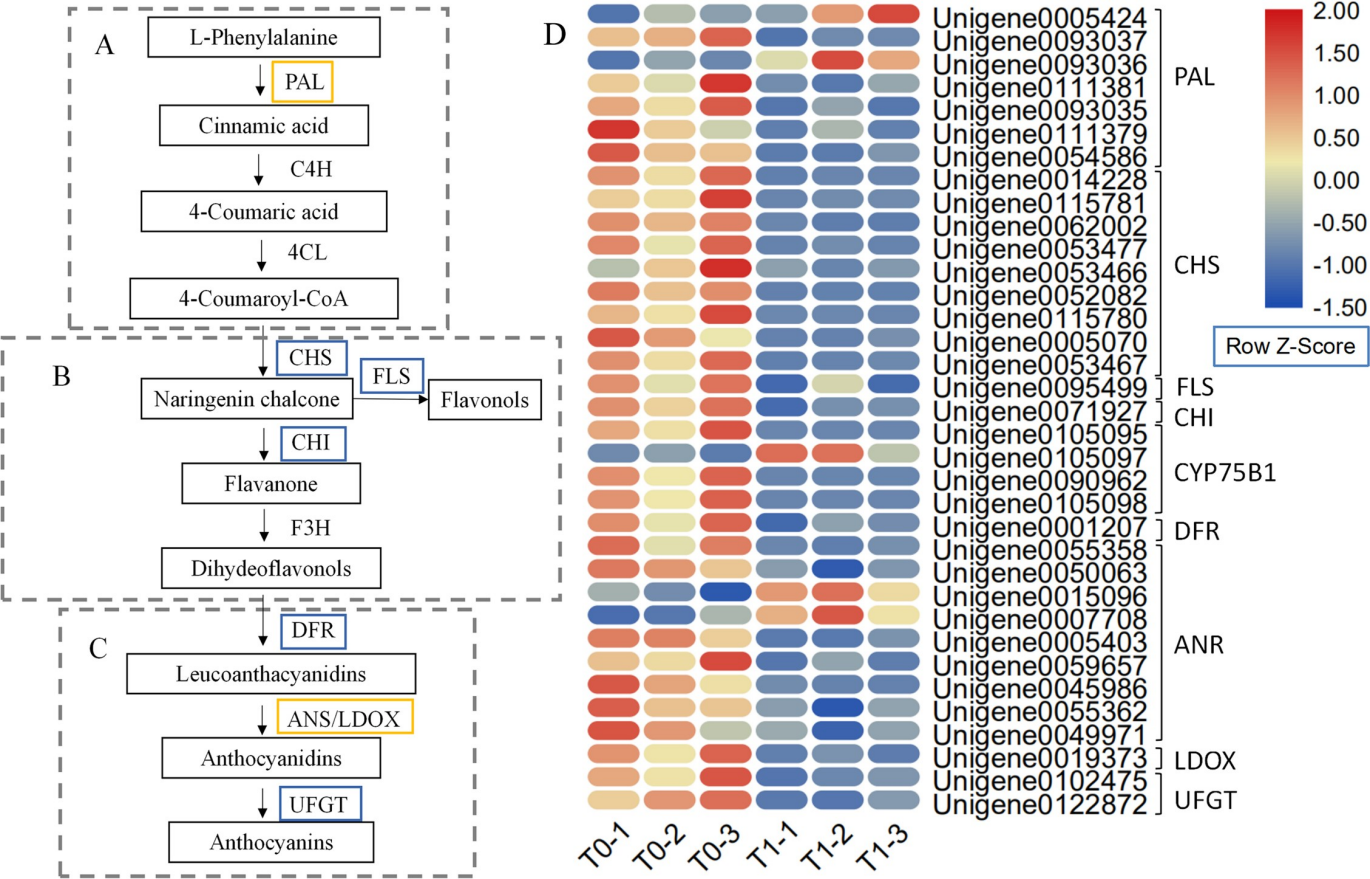
Anthocyanin biosynthesis pathway and heat map. The first (A), second (B), and third (C) stages of anthocyanin biosynthesis [31, 32]. The blue box indicates downregulated genes, and the orange box indicates both upregulated and downregulated genes.

The biosynthesis of the leaf pigment anthocyanin is a very complex process that includes several metabolic pathways, including phenylalanine (Fig 6A), flavonoid (Fig 6B), and anthocyanin (Fig 6C) metabolic pathways [31, 32]. Based on the pathway analysis of the DEGs, a total of 31 key enzyme-encoding genes may be involved in the synthesis and accumulation of anthocyanins in *P*. *setaceum* ‘Rubrum’ leaves. Of the seven genes annotated to the PAL family, only two genes were upregulated (Fig 6D). Nine genes annotated to chalcone synthase (CHS), one gene annotated to CHI, one gene annotated to flavonol synthase (FLS), and one gene annotated to DFR were downregulated. Within the anthocyanidin reductase (ANR) family, which has nine annotated genes, only two genes were upregulated in the T1 stage. One gene annotated to leucoanthocyanidin dioxygenase (LDOX) was downregulated (Fig 6D). In the anthocyanin metabolism pathway, two downregulated genes were annotated to the UFGT family downstream of anthocyanin biosynthesis (Fig 6D).

We predicted the genes encoding three protein families of anthocyanin synthesis, namely R2R3-MYB, bHLH and WDR, and four genes were predicted to be involved in *P*. *setaceum* ‘Rubrum’ leaf color formation (Table 5). A total of 34 genes were differentially expressed in T0 and T1 stages, two of which were R2R3-MYB TFs and related to anthocyanin. Among them, the expression of *MYB1* was upregulated, resulting in the inhibition of anthocyanin synthesis. The annotation of the *C1* gene indicated it encodes an anthocyanin regulatory protein whose downregulation promotes anthocyanin synthesis. A total of 33 differentially expressed genes were predicted in the bHLH TF family, and two genes that were related to anthocyanin were annotated as *R-S*: one was upregulated, while the other one was downregulated. From a total of 43 genes from the WD40 family of TFs, four were differentially expressed and downregulated in T1, but none of these genes were related to anthocyanins. These results suggested that the leaf color in *P*. *setaceum* ‘Rubrum’ may be jointly regulated by a complex formed by bHLH and R2R3-MYB.

**Table 5:** Differentially expressed transcription factors associated with anthocyanin biosynthesis in *Pennisetum setaceum* ‘Rubrum’.

Transcription factor family	Gene ID	log2 Ratio (T1/T0)	Symbol	Gene annotation
MYB	Unigene0053710	1.10	MYB1	R2R3-MYB transcription factor MYB4 [*Cenchrus purpureus*]
	Unigene0012779	-5.71	C1	Anthocyanin regulatory C1 protein [*Dichanthelium oligosanthes*]
bHLH	Unigene0063953	1.67	R-S	PREDICTED: anthocyanin regulatory R-S protein-like [*Setaria italica*]
	Unigene0063954	-1.08	R-S	PREDICTED: anthocyanin regulatory R-S protein-like [*Setaria italica*]

### Real-time quantitative PCR validation of genes identified in the *P*. *setaceum* ‘Rubrum’ transcriptome

**Fig 7:**
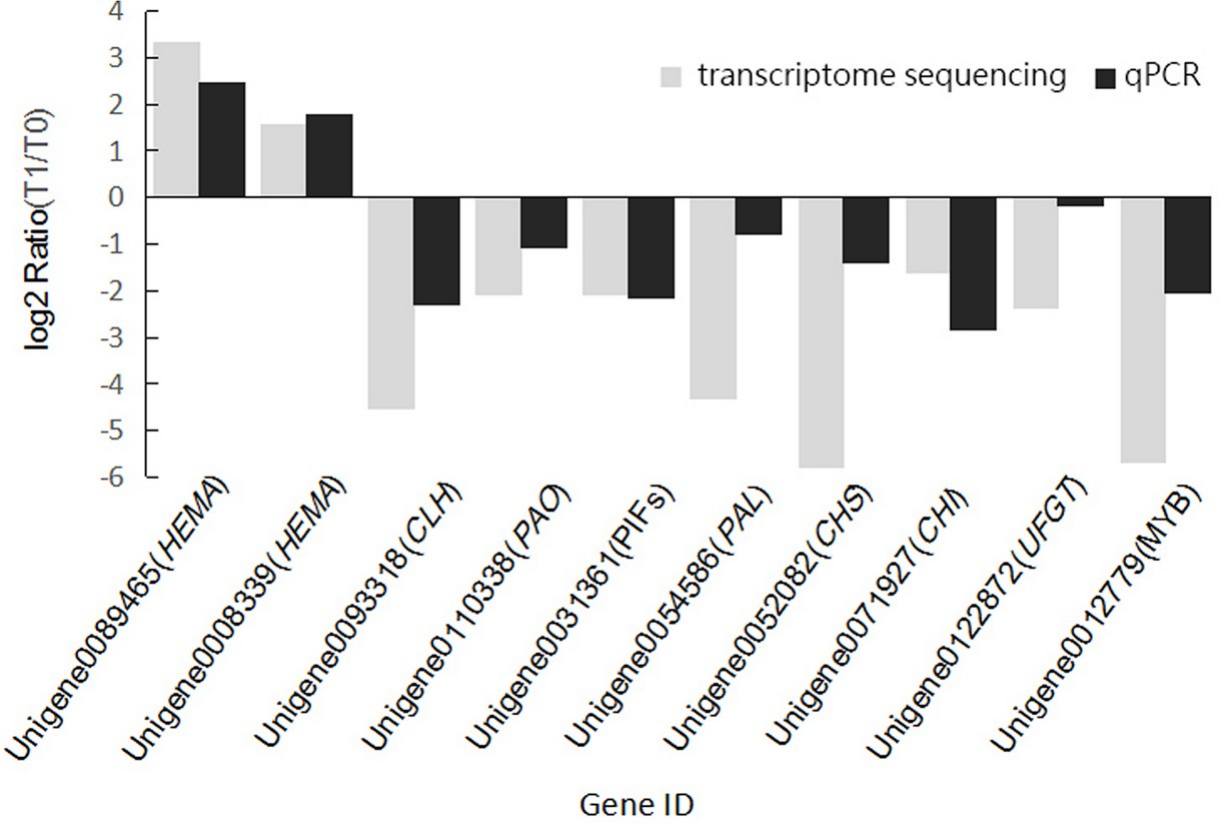
Real-time PCR validation of transcriptome sequencing results. The log2 Ratio(T1/T0) represents the relative gene expression.

To further verify the reliability of the transcriptome sequencing, we randomly selected 10 DEGs closely related to chlorophyll metabolism and anthocyanin metabolism for verification. *MDH* and *ACP* were selected as internal reference genes based on the results of the gene expression study on *Pennisetum glaucum* [33]. The dissolution curves of the 12 genes were unimodal. The results of qPCR detection of these genes in samples from T0 and T1 were consistent with the upregulated and downregulated expression in the transcriptome dataset (Fig 7), confirming the reliability of the transcriptomic sequencing.

## Discussion

### KEGG pathways for chlorophyll and anthocyanin biosynthesis are associated with leaf color changes in response to light condition

Transcriptome sequencing is a highly efficient and affordable method for the fast generation of nearly all cellular transcripts under different physiological conditions. It has been widely used in studies using *A*. *thaliana*, maize, and rice, as well as ornamental plants such as *Lagerstroemia* [34] and *Lilium* ‘Tiny Padhye’ [35].

The KEGG database is used to analyze the metabolic pathways and functions of gene products to elucidate the complex biological behaviors of genes. The KEGG enrichment analysis conducted in the present study was consistent with our previous results [3]. The red-greenness a* values of the color index decreased significantly in the T1 stage, when the leaf color reached a blue-yellowness grade. Physiological experiments showed increased chlorophyll content at T1 stage [3], suggesting that photosynthesis was enhanced, which was verified with the results that showed the upregulated DEGs were mainly enriched in KEGG pathways related to chlorophyll and photosynthesis. Similarly, the downregulated DEGs were enriched in the pathways related to anthocyanin biosynthesis in the T1 stage, indicating a decrease in anthocyanin content, which was consistent with the previous leaf color parameters and physiological experimental results [3].

### Expression of genes related to chlorophyll metabolism regulate leaf color changes in response to light

Chlorophyll is the pigment responsible for the green color of plant leaves, and its biosynthesis is well studied [5, 6]. HemA is the first enzyme of the chlorophyll biosynthesis pathway and catalyzes the transformation of L-glutamyl-tRNA to δ-glutamyl RNA [6]. *HemC* genes encode porphobilinogen deaminase, which functions in the second stage of chlorophyll biosynthesis. In the last stage of chlorophyll biosynthesis, Mg-chelatase is the critical enzyme at the branching point in the chlorophyll biosynthesis pathway. The three subunits of the Mg-chelatase enzyme are magnesium chelatase subunit ChlD, magnesium chelatase subunit ChlH, and magnesium-chelatase subunit ChlI [36]. After the shading treatment, the *P*. *setaceum* ‘Rubrum’ leaves turned green. We found that the expression of six *HemA*, two *HemC*, three *ChlD*, two *ChlH*, one *ChlI*, five *POR*, one *ChlG*, and one *CAO* gene was upregulated in the T1 stage, which resulted in an increase in the chlorophyll content of the leaves, promoting their green coloration. This finding was consistent with the results of previous physiological experiments in which chlorophyll content in the T1 stage was significantly higher than that in the T0 stage, consistent with the results of leaf color parameters [3]. These results all suggest the expression of genes related to chlorophyll synthesis is sensitive to light intensity and affects the dynamic color change observed in *P*. *setaceum* ‘Rubrum’ leaves.

The above genes that play a key role in chlorophyll synthesis have been studied in other plants. A study on yellow leaves of *Lagerstroemia indica* found that downregulation of genes such as *HemA* and *HemD* hindered normal chlorophyll biosynthesis [37]. Sakuraba et al. studied faded green leaf mutants in rice and found that *POR* is an essential gene for chlorophyll biosynthesis in rice leaves [38]. The literature shows that *HemA*, *HemD*, and *POR* are critical genes in chlorophyll biosynthesis. Combined with the results of previous studies, the results presented herein confirm the reliability of our conjecture about the key genes in the *P*. *setaceum* ‘Rubrum’ chlorophyll synthesis process.

In the chlorophyll degradation process, *NYC1* and *NOL* catalyze the degradation of chlorophyll *b* to chlorophyll *a* [39], while the CLH enzyme catalyzes the removal of the phytol from chlorophyll *a* to produce chlorophyllide [40]. PaO is another enzyme involved in the chlorophyll degradation pathway. Our analysis identified 10 DEGs that encode enzymes related to chlorophyll degradation, namely one *NOL*, one *NYC1*, three *CHL*, and five *PaO* genes. These genes were significantly downregulated in the T1 stage and the degradation rate of chlorophyll was reduced. Consequently, the chlorophyll content in leaves from the T1 stage was greater than that in stage T0. These results were consistent with previous leaf color parameters and physiological observations [3], further suggesting these genes are related to chlorophyll degradation and leaf color changes in *P*. *setaceum* ‘Rubrum’ in response to changes in light conditions.

Previous studies have explored the degradation of chlorophyll in different plants. Sato et al. studied the chlorophyll degradation pathway during rice senescence and found that *nol* and *nyc1* rice mutants showed a stay-green phenotype and chlorophyll *b* degradation was severely inhibited, which indicated that *NOL* and *NYC1* promoted chlorophyll degradation [39]. The results of present study show that at the T1 stage, one *NYC1* was downregulated to promote chlorophyll degradation, while one *NYC1-LIKE* (*NOL*) gene was upregulated to inhibit chlorophyll degradation, indicating that the regulation of *NOL* on leaf color may be more complex in *P*. *setaceum* ‘Rubrum’. Takamiya et al. showed that both CLH and PaO are essential enzymes for chlorophyll degradation in plants [30], while Schenk et al. found that the chlorophyllases genes *AtCLH1* and *AtCLH2* are not required for chlorophyll decomposition during leaf senescence in *A*. *thaliana* [41]. The results of the present study suggest that *CLH* and *PaO* expression is related to chlorophyll degradation and that their gene products are likely necessary for changes in *P*. *setaceum* ‘Rubrum’ leaf color.

Among the TFs related to chlorophyll biosynthesis, five regulatory genes that are members of the PIFs family were predicted to be related to chlorophyll degradation. In the PIFs family, we predicted that the helicases/helix genes would be downregulated as chlorophyll content increased, suggesting that these genes promote chlorophyll degradation. Huq et al. reported a negative correlation between PIF1 and chlorophyll biosynthesis in plants [42], and Shin et al. found that PIF3 reduced the rate of chlorophyll biosynthesis and photosynthetic efficiency by inhibiting the expression of *GUN5* and other essential genes for chlorophyll biosynthesis in the dark [43]. We found that under shading conditions, the downregulation of PIFs promotes the synthesis of chlorophyll. Previous research and our results show that the PIFs family can regulate the biosynthesis of chlorophyll.

### Genes differentially regulated by light are related to anthocyanin metabolism

PAL is an essential enzyme for the first stage of anthocyanin biosynthesis, where it catalyzes L-phenylalanine to form *trans-*cinnamic acid [31]. CHS and CHI are two important enzymes in the flavonoid biosynthesis pathway. Flavonols are a side product of naringenin chalcone produced in a reaction catalyzed by FLS [32]. The DFR enzyme can use dihydroflavonols, dihydromyricetin, dihydrokaempferol, and/or dihydroquercetin as substrates to synthesize leucoanthocyanidins [11]. After the shading treatment, the leaf color of *P*. *setaceum* ‘Rubrum’ changed from purple to green, which correlated with the significant downregulation of anthocyanin biosynthesis genes. These included seven *PAL*, nine *CHS*, one *FLS*, one *CHI*, one *DFR*, nine *ANR*, one *LDOX*, and two *UFGT* genes. The transcriptome sequencing data for anthocyanin biosynthesis pathway members showed that most of these genes were downregulated in the T1 stage, which was consistent with results from physiological experiments on flavonoid content, anthocyanin content, PAL enzyme activity, and CHI enzyme activity previously reported [3]. When the genes encoding key enzymes in the anthocyanin biosynthesis pathway were downregulated, the leaves in the T1 stage lacked purple or red coloration, which was also consistent with previous leaf color parameter results that confirmed the purplish-red leaves in the T0 stage and green leaves in the T1 stage [3].

Similar conclusions were reported in studies on flower and pericarp color. For example, Sun et al. found that the expression pattern of *FhCHS1* in *Freesia hybrida* flowers was significantly related to the accumulation patterns of anthocyanins during flower development [44]. Furthermore, the function of *FhCHS1* was verified by ectopic expression in *Petunia hybrida*, and it was found that *CHS1* was responsible for the flower color transition from the original white to pink [44]. Christopher et al. reported higher anthocyanin content in orange than in yellow *Clivia miniata*, and observed the same trend for the expression of *CHS* and *DFR* [45]. Zhao et al. found that the transcription of FLS was downregulated throughout apple fruit development under reduced UV [46]. Zhao et al. showed that the red peel color of *Litchi chinensis* fruits is attributed to *UFGT* and its role in anthocyanin biosynthesis [47]. Therefore, we believe that these genes play an important role in the purple leaf phenotype of *P*. *setaceum* ‘Rubrum.’

The MYB-bHLH-WD40 (MBW) transcription complexes mainly regulate anthocyanin biosynthesis [13, 14]. However, the results of our study showed that during the process of leaf color change from purple to green in *P*. *setaceum* ‘Rubrum,’ the DEGs related to anthocyanins were not annotated with WD40, whereas the expression of anthocyanin genes in MYB and bHLH TFs changed significantly. Therefore, we hypothesized that the purple leaf color of *P*. *setaceum* ‘Rubrum’ is determined by the complex of bHLH and MYB family TFs. In our study, *C1* of the R2R3-MYB gene family and one *R-S* gene from the bHLH gene family were downregulated, which was positively correlated with anthocyanin synthesis. Shin et al. expressed maize *C1* and *R-S* genes in rice endosperm, and the resulting kernels produced several different kinds of flavonoids [48], which was similar to our results. Therefore, we hypothesized that *C1* and *R-S* can activate structural genes in the flavonoid biosynthesis pathway individually or synergistically. However, our sequencing data showed that only one *R-S* gene in the bHLH gene family was upregulated, suggesting its role in the inhibition of anthocyanin synthesis. This may be because the R2R3-MYB gene family plays a key role in the regulation of anthocyanin synthesis mediated by the MBW complex [49]. Hence, the upregulated expression of one *R-S* gene has a limited effect on anthocyanin synthesis, although the specific mechanisms of these gene products warrant further study. The expression of *MYB1* from the MYB family of TFs was upregulated after the leaf color turned green, indicating that *MYB1* may negatively regulate anthocyanin synthesis in *P*. *setaceum* ‘Rubrum.’ Most of the R2R3-MYB genes in *Arabidopsis* positively regulate anthocyanin synthesis, while a few genes are negative regulators of this pathway [50]. For example, Schwinn found that *MYB1* positively regulates anthocyanin biosynthesis in onion [51], which is contradictory to the results of the present study. Thus, there may exist different mechanisms in different species by which *MYB1* regulates anthocyanin biosynthesis.

## Conclusions

In this study, Illumina RNA-Seq was used to conduct transcriptome sequencing analysis, which was used to assemble 93,750 unigenes. Genetic analysis based on the Illumina sequencing was used to identify DEGs that revealed many candidate genes related to leaf color, including genes involved in the metabolic pathway of chlorophyll and anthocyanins. This transcriptome analysis provides important information for the continued study of leaf color-related genes and offers not only a basis for future functional genomics research in *P*. *setaceum* ‘Rubrum,’ but also resources for genomics research in other *P*. *setaceum* cultivars. The study is of considerable significance for horticulture practices and can help to improve the ornamental quality of *P*. *setaceum* ‘Rubrum.’

## Supporting information

S1 Fig Saturation of six samples of *Pennisetum setaceum* ‘Rubrum’. (TIF)

S2 Fig Statistics of gene coverage for six samples of *Pennisetum setaceum* ‘Rubrum’. (TIF)
