# Supplementary figures and images for: Screening of key genes responsible for Pennisetum setaceum ‘Rubrum’ leaf color using transcriptome sequencing
Source: PLoS One. 2020 Nov 23;15(11):e0242618. doi: 10.1371/journal.pone.0242618 (PMC7682885; doi:10.1371/journal.pone.0242618)

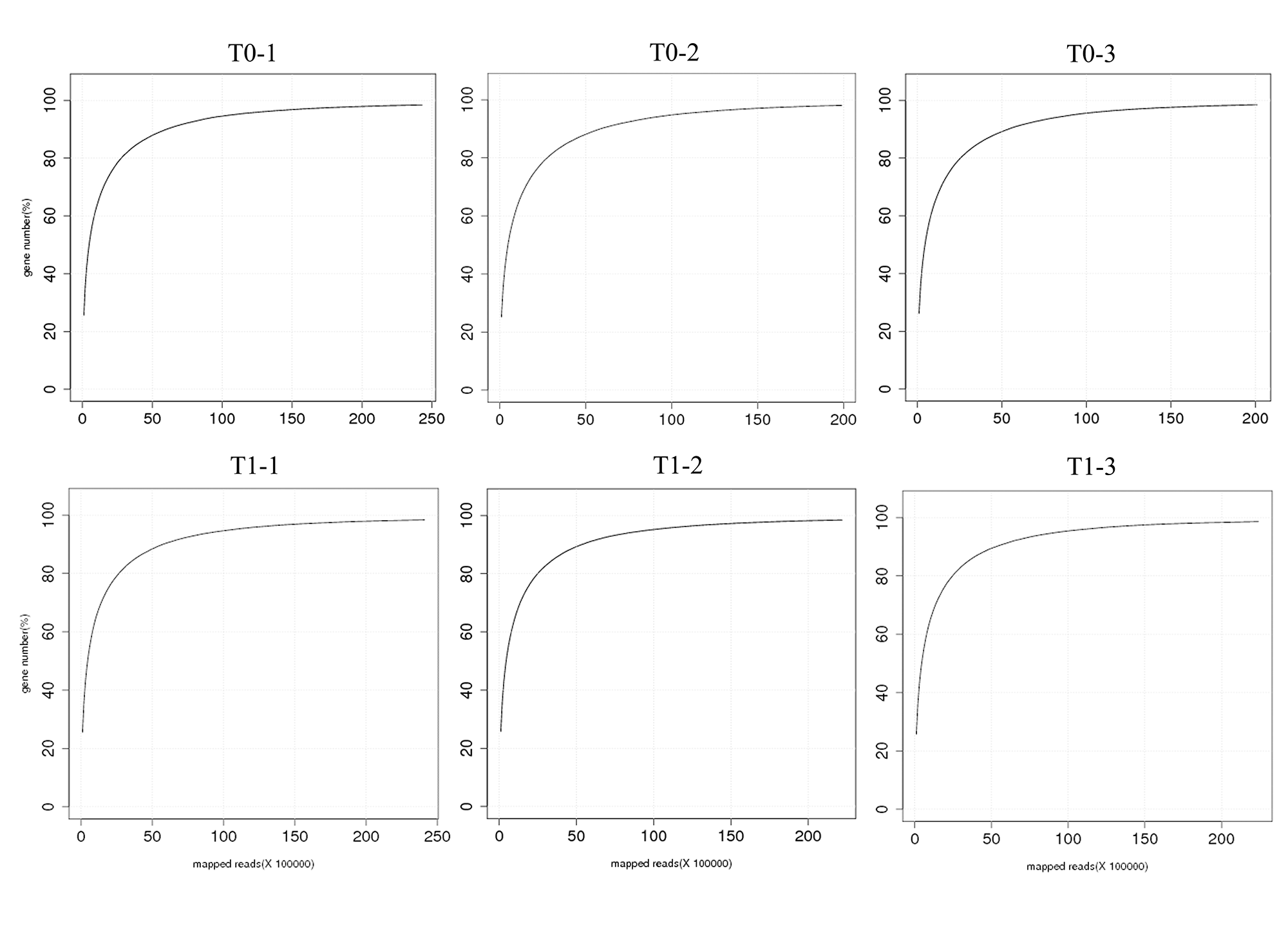

Supplement: S1 Fig — (TIF) [file pone.0242618.s001.tif]

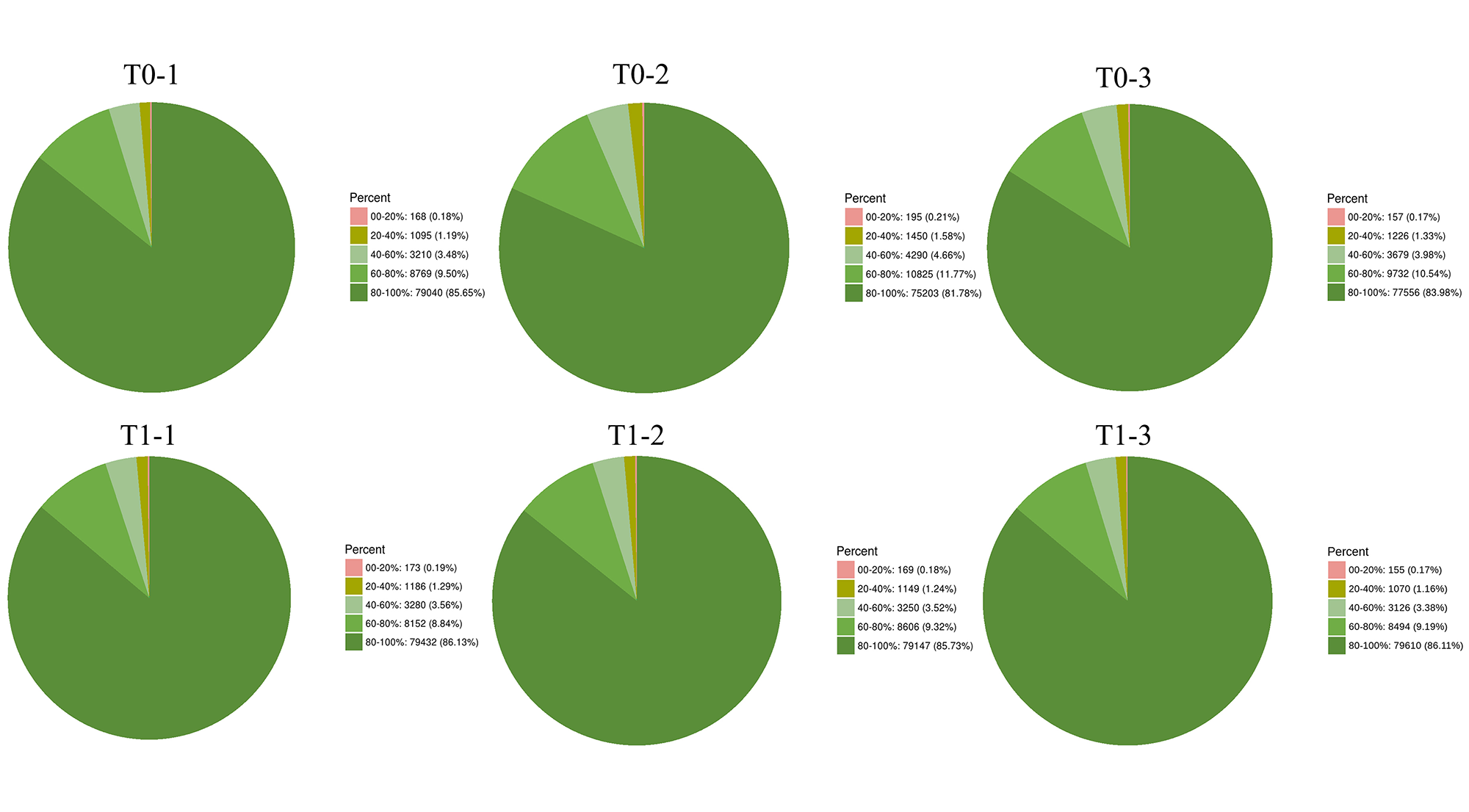

Supplement: S2 Fig — (TIF) [file pone.0242618.s002.tif]
